# MRI prospective survey on cardiac and hepatic iron in transfusion-dependent thalassemia intermedia patients treated with desferrioxamine, deferiprone and deferasirox

**DOI:** 10.1186/1532-429X-17-S1-P357

**Published:** 2015-02-03

**Authors:** Antonella Meloni, Mari Giovanna Neri, Maria Chiara Resta, Massimiliano Missere, Antonino Vallone, Vincenzo Positano, Silvia Macchi, Crocetta Argento, Daniele De Marchi, Alessia Pepe

**Affiliations:** 1 CMR Unit, Fondazione G. Monasterio CNR-Regione Toscana, Pisa, Italy; 2 Struttura Complessa di Radiologia, OSP. SS. Annunziata ASL Taranto, Taranto, Italy; 3 Dipartimento di Radiologia, Un. Cattolica del Sacro Cuore - Centro di Ricerca e Formazione ad Alta Tecnologia "G. Paolo II", Campobasso, Italy; 4 Istituto di Radiologia, Az. Osp. "Garibaldi" Presidio Ospedaliero Nesima, Catania, Italy; 5 Servizio trasfusionale, Ospedale Santa Maria delle Croci, Ravenna, Italy; 6 Centro di Talasssemia, Ospedale San Giovanni Di Dio, Agrigento, Italy

## Background

Few studies have evaluated the efficacy of iron chelation therapy in thalassemia intermedia (TI) patients. Our study aimed to prospectively assess by quantitative Magnetic Resonance imaging (MRI) the efficacy of the three available chelators in monotherapy in transfusion dependent (TD) TI patients.

## Methods

Among the 325 TI patients enrolled in the MIOT (Myocardial Iron Overload in Thalassemia) network, we selected 103 TI patients TD with an MRI follow-up (FU) study at 18±3 months who had been received one chelator alone between the two MRI scans. Iron overload was assessed by the T2* multiecho technique. Hepatic T2* values were converted into liver iron concentration (LIC) values.

## Results

Three groups of patients were identified: 27 patients (13 females, mean age 40.12±10.31 years) treated with desferioxamine (DFO - mean dosage 37.52±8.69 mg/kg/die), 23 patients (14 females, mean age 34.73±10.67 years) treated with deferiprone (DFP- dosage 71.70±14.46mg/kg/die) and 14 patients (9 females, mean age 36.63±10.92 years) treated with deferasirox (DFX - mean dosage 27.75±5.04 mg/kg/die). Excellent/good levels of compliance were similar in the DFO (92.6%), DFP (100%) and DFX (100%) groups (P=0.345). The mean starting age of regular transfusion was 14.73±15.89 years.

At baseline in DFO group two patients (7.4%) showed a global heart T2*<20 ms and one of them showed no cardiac iron at the FU. At baseline in DFP group two patients (8.7%) showed a global heart T2*<20 ms and one of them showed no cardiac iron at the FU. All the 5 patients (35.7%) under DFX therapy with pathological global heart T2* at the baseline remained at the same status at the FU. The percentage of patients who maintained a normal global heart T2* value was comparable for DFO (100%), DFP (100%) and DFX (88.9%) groups (P=0.164).

Among the 46 patients with hepatic iron at baseline (MRI LIC ≥3 mg/g/dw), the reduction in the MRI LIC values was significant only in the DFO group (DFO: -3.39±6.38 mg/g/dw P=0.041; DFP: -2.25±6.01 mg/g/dw P=0.136 and DFX: -0.36±5.56 mg/g/dw P=0.875). The decrease in MRI LIC values was comparable among the groups (P=0.336). The number of patients with a MRI LIC<3 mg/g/dw went up from 10 (37%) to 11 (40.7%) in the DFO group, from 6 (26.1%) to 8 (34.8%) in the DFP group and from 2 (14.3%) to 8 (57.1%) in the DFX group. The percentage of patients who maintained a normal MRI LIC value was comparable for DFO (90%) vs DFP (50%) and DFX (100%) groups (P=0.191).

## Conclusions

Prospectively in transfusion-dependent TI patients at the dosages used in the clinical practice, DFO and DFP showed 100% efficacy in maintaining a normal global heart T2* value while DFX had 100% efficacy in maintaining a normal LIC value.

Further prospective studies involving more patients with iron at the baseline are needed to establish which is the most effective drug in reducing iron levels.

## Funding

The MIOT project receives "no-profit support" from industrial sponsorships (Chiesi Farmaceutici S.p.A. and ApoPharma Inc.).

**Figure 1:**
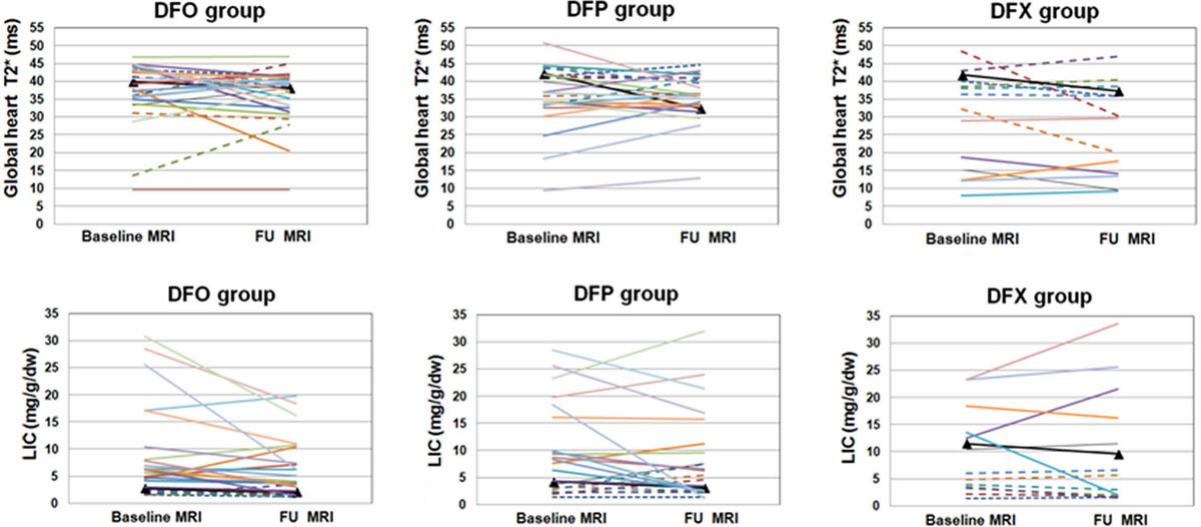
Changes of global haert T2* (up) and MRI LIC values (bottom) in TD TI patients treated with different iron chelators in monotherapy

